# Maximum velocity and leg-specific ground reaction force production change with radius during flat curve sprinting

**DOI:** 10.1242/jeb.246649

**Published:** 2024-02-28

**Authors:** Gabriela B. Diaz, Ryan S. Alcantara, Alena M. Grabowski

**Affiliations:** ^1^ Applied Biomechanics Lab, University of Colorado Boulder, Department of Integrative Physiology, Boulder, CO 80309, USA; ^2^ Department of Veterans Affairs, Eastern Colorado Healthcare System, Denver, CO 80205-3540, USA

**Keywords:** Running, Biomechanics, Performance, Bend-running

## Abstract

Humans attain slower maximum velocity (*v*_max_) on curves versus straight paths, potentially due to centripetal ground reaction force (GRF) production, and this depends on curve radius. Previous studies found GRF production differences between an athlete's inside versus outside leg relative to the center of the curve. Further, sprinting clockwise (CW) versus counterclockwise (CCW) slows *v*_max_. We determined *v*_max_, step kinematics and individual leg GRF on a straight path and on curves with 17.2 and 36.5 m radii for nine (8 male, 1 female) competitive sprinters running CW and CCW and compared *v*_max_ with three predictive models. We combined CW and CCW directions and found that *v*_max_ slowed by 10.0±2.4% and 4.1±1.6% (*P*<0.001) for the 17.2 and 36.5 m radius curves versus the straight path, respectively. *v*_max_ values from the predictive models were up to 3.5% faster than the experimental data. Contact length was 0.02 m shorter and stance average resultant GRF was 0.10 body weights (BW) greater for the 36.5 versus 17.2 m radius curves (*P*<0.001). Stance average centripetal GRF was 0.10 BW greater for the inside versus outside leg (*P*<0.001) on the 36.5 m radius curve. Stance average vertical GRF was 0.21 BW (*P*<0.001) and 0.10 BW (*P*=0.001) lower for the inside versus outside leg for the 17.2 and 36.5 m radius curves, respectively. For a given curve radius, *v*_max_ was 1.6% faster in the CCW compared with CW direction (*P*=0.003). Overall, we found that sprinters change contact length and modulate GRFs produced by their inside and outside legs as curve radius decreases, potentially limiting *v*_max_.

## Teaser

**Summary:** Sprinters decrease contact length and modulate ground reaction forces produced by their inside and outside legs as curve radius decreases, potentially limiting maximum velocity when sprinting along flat curves.

## INTRODUCTION

Attaining maximum sprinting velocity (*v*_max_) while running is particularly important in a variety of circumstances such as predator–prey relationships as well as athletic competitions. As running is rarely along a straight path, being able to maintain *v*_max_ while on curves in addition to on a straight path is advantageous. While some animals such as greyhounds and cheetahs retain their *v*_max_ on curves compared with straight paths (Usherwood and Wilson, 2005a; Wilson et al., 2013), other animals such as horses (Tan and Wilson, 2011) and humans have slower *v*_max_ on a curve relative to a straight path (Jain, 1980; Greene, 1985; Chang and Kram, 2007; Churchill et al., 2015, 2016). In humans the underlying biomechanics that affect *v*_max_ on a curve have not been fully explored. Additionally, studying curved sprinting in track and field athletes is specifically relevant to outdoor athletics events such as the 200 m and 400 m sprint, where more than half the race is run on a flat (unbanked) curve (Meinel, 2008).

**List of abbreviations**

*B*: unstandardized model coefficients
BW: body weight
CCW: counterclockwise
cGRF: centripetal ground reaction force
CW: clockwise
***g***: acceleration due to gravity
GCS: global coordinate system
GRF: ground reaction force
*L*_c_: contact length
LMEM: linear mixed effects model
*r*: curve radius
rGRF: resultant ground reaction force
*t*_a_: aerial time
*t*­_step_: step time
*t*_swing_: swing time
vGRF: vertical ground reaction force
*v*_max_: maximum sprinting velocity

Track and field athletes must be able to run on a range of different curve radii due to lane assignment or track design. Regulation athletic track curve radii can range from 17.2 m (innermost lane of a regulation 200 m indoor track) to 45.0 m (outermost lane of a regulation 400 m outdoor track) (Meinel, 2008). Additionally, the attenuation of *v*_max_ in humans depends on the curve radius, such that running on smaller curve radii results in a slower *v*_max_ than that on larger curve radii (Jain, 1980; Greene, 1985; Chang and Kram, 2007). Previous studies have measured *v*_max_ of athletes who performed maximum effort sprints on straight and on counterclockwise (CCW) track curves with a radius equivalent to lane 2 of a 400 m track (37.72 m) and lane 1 of a 200 m track (17.2 m) and found that *v*_max_ was 2.3–4.7% slower on a 37.72 m radius curve compared with a straight path (Churchill et al., 2015, 2016) and 8.9% slower on a 17.2 m curve compared with a straight path (Taboga et al., 2016).

To predict performance in athletics, previous studies have proposed mathematical models to predict curve-running *v*_max_ in humans (Jain, 1980; Usherwood and Wilson, 2005b). McMahon (1984) developed a mathematical model to predict curve-running *v*_max_ on flat curves for a range of radii (*R*) that uses straight-running *v*_max_ (*V*_max_), and kinematic variables such as aerial time (*t*_a_­), contact length (*L*_c_) and step time (*t*_step_) (Eqn 1):
(1)

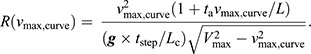
This model assumed that *L*_c_ and step frequency are constant and independent of curve radius. *L*_c_ is the distance an athlete moves forward during ground contact, and step frequency is the inverse of time from heel-strike to contralateral heel-strike. The model predictions were compared with experimental data from one subject who ran on a turf surface at five different curve radii (approximately 3–30 m) and well-predicted *v*_max_ for larger curve radii, but over-predicted *v*_max_ for radii <15 m. Greene (1985) simplified the mathematical model proposed by McMahon (1984) to include only *v*_max_ and local gravitational acceleration (***g***) (Eqn 2):
(2)

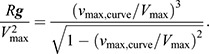
This model assumed a constant resultant ground reaction force (GRF) produced by each leg at *v*_max_ that was independent of curve radius. The model predictions from Greene (1985) were compared with experimental data from 10 and 13 runners who ran on flat grass and concrete surfaces, respectively, at five different curve radii (approximately 3–30 m) and over-predicted *v*_max_ for a given curve radius regardless of whether runners were sprinting on a grass or concrete surface. Additionally, Usherwood and Wilson (2005b) developed a model to predict curve-running *v*_max_ on a banked 200 m indoor track (Eqn 3):
(3)

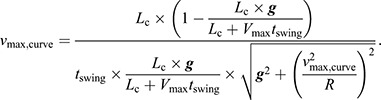
This model also assumed that *L*_c_ and the maximum resultant GRF produced by each leg are constant and independent of curve radius. They also assumed that leg swing time (*t*_swing_) is constant, and that step frequency decreases with curve radius. The model developed by Usherwood and Wilson (2005b) predicts curve-running *v*_max_ from straight-running *v*_max_, *t*_swing_ and *L*_c_. They calculated straight-running *v*_max_ for men and women from the average times published for all (heats, quarterfinals, semi-finals and finals) 200 m outdoor sprint races at the 2004 Olympic Games to predict­ the 2004 World Indoor Championship race times. The model well-predicted the men's 200 m indoor race times, but underpredicted the 200 m indoor women's race times.

The assumptions that underly each model that predict *v*_max_ attenuation on curves versus a straight path may affect the model results compared with experimental data. Previous experimental studies have found that step length is independent of curve radius for 3–30 m radii (McMahon and Greene, 1979; Greene, 1985). However, more recent experimental studies have found shorter step lengths during curve running at *v*_max_ on radii of 1–6 m, 17.2 m and 37.5 m compared with running on a straight path (Chang and Kram, 2007; Taboga et al., 2016; Churchill et al., 2015). Moreover, previous experimental studies have found an increase in contact time with decreasing curve radii for 1–6 m radius curves, but no difference in step frequency (Chang and Kram, 2007) and a 2.4% reduction in step frequency during curve running at *v*_max_ on a 17.2 m radius curve compared with a straight path (Taboga et al., 2016). Finally, curve-running *v*_max_ may not be limited by the magnitude of the resultant GRF. Though Usherwood and Wilson (2005b) found that the maximum resultant GRF did not change and accounted for the slower *v*_max_ for an indoor 200 m race on a banked curve, Chang and Kram (2007) found that the maximum resultant GRF produced during *v*_max_ on flat curves with 1–6 m radii was lower than that produced during *v*_max_ on a straight path. Thus, there may be other physiological limitations that result in the *v*_max_ attenuation on curves compared with a straight path. Lastly, an implied assumption in all three models that predict *v*_max_ on curves is that the two legs exhibit the same biomechanics. However, previous studies have found that while running CCW at *v*_max_ on a 37.5 m radius curve, step length was shorter for the outside leg but not for the inside leg compared with a straight path, and step frequency was slower for the outside leg but not for the inside leg compared with a straight path (Churchill et al., 2015). Thus, to better understand potential performance implications during athletics sprints, we measured *v*_max_ on a straight path and two flat curves with radii representative of lane 1 of a regulation 200 m and 400 m track and compared these with the curve-running *v*_max_ predicted from the three mathematical models (Eqns 1, 2 and 3). We also measured kinematic variables and GRFs from the inside and outside legs relative to the center of the curve when sprinters ran at *v*_max_ on these curve radii to determine the leg-specific biomechanical changes during curve-running *v*_max_.

Sprinting on a curve requires an athlete to produce centripetal ground reaction forces (cGRFs) that accelerate their body towards the inside of the curve, where maintaining a given velocity for a smaller curve radius requires greater cGRF. In the case of flat curves, cGRF equals the product of a sprinter's mass and forward velocity squared divided by curve radius. Experimental data indicate that the leg on the inside and outside of a curve relative to the center of the curve may have unique roles in producing the cGRF needed to navigate a curve at a particular velocity (Chang and Kram, 2007; Churchill et al., 2016; Judson et al., 2019). Leg-specific cGRF production may change with curve radius, as prior studies suggest that the inside leg produces greater cGRF than the outside leg on a 37.72 m radius curve (Churchill et al., 2016) but produces lower cGRF than the outside leg on 1–6 m radius curves (Smith et al., 2006; Chang and Kram, 2007). Additionally, vertical GRF (vGRF) production is similar for the inside and outside leg on a 37.72 m radius curve (Churchill et al., 2016), but the inside leg produces lower vGRF than the outside leg on 1–6 m radius curves (Chang and Kram, 2007). Thus, we measured and compared leg-specific cGRF and vGRF production across intermediate curve radii (17.2 m and 36.5 m) to potentially identify the underlying mechanisms limiting *v*_max_ on flat curves.

Modern athletics events that include curves (≥200 m) are completed in the CCW direction and sprinters train to run along curves in the CCW direction. Experimental data support the existence of a potential biomechanical training effect of curve-running direction, as *v*_max_ slows by 1.9% when sprinting on a curve with a 17.2 m radius in the clockwise (CW) compared with the CCW direction (Taboga et al., 2016). Therefore, sprinting direction may also affect *v*_max_, kinematic variables and GRFs, and depend on curve radius. Thus, we quantified the effect of sprinting direction on *v*_max_, kinematic variables and GRFs to inform future work aimed at determining leg-specific biomechanics in populations with apparent biomechanical asymmetries (e.g. athletes with a unilateral lower-leg amputation).

We analyzed maximum effort sprinting and the corresponding changes in *v*_max_, kinematic variables and GRF production of athletes on a straight path and on CCW and CW curves representative of the innermost lane of a flat (unbanked) 200 m and 400 m regulation athletics track (17.2 m and 36.5 m radii). In line with previous studies (Churchill et al., 2015, 2016; Taboga et al., 2016) and mathematical models (Greene, 1985; McMahon, 1984; Usherwood and Wilson, 2005a,b), we hypothesized that during maximum effort sprinting: (1) *v*_max_ would be slower on the 17.2 m and 36.5 m radius curves relative to a straight path regardless of curve sprinting direction, (2) mathematical models (Eqns 1, 2 and 3; McMahon, 1984; Greene, 1985; Usherwood and Wilson, 2005a,b) would overpredict curve-running *v*_max_, (3) *L*_c­_, step frequency and swing time would differ between curve radii and between the inside and outside legs, (4) stance-average resultant GRF (rGRF_avg_) would not change between curve radii or between the inside and outside leg, (5) the inside leg would produce greater stance-average cGRF (cGRF_avg_) than the outside leg, but the outside leg would produce greater stance-average vGRF (vGRF_avg_) than the inside leg on both curve radii, and (6) *v*_max_ would be slower on curves in the CW versus CCW direction.

## MATERIALS AND METHODS

### Study population

A convenience sample of 9 National Collegiate Athletic Association (NCAA) track and field athletes (8 male, 1 female; 200 m personal best: 22.60±2.39 s; 400 m personal best: 47.76±1.49 s; body mass: 74.6±9.5 kg; height: 1.83±0.10 m; age: 21±1 years, means±s.d.) with curve sprinting experience participated. We used data from Chang and Kram (2007) to estimate an appropriate sample size for peak resultant GRF, step length and step frequency between inside and outside legs, and maximum velocity for running on a straight path compared with a 6 m radius curve. We set *P*=0.05, used a paired *t*-test design, and found significant effect sizes in peak resultant GRF (0.75), step length (0.98), step frequency (0.95) and maximum velocity (1.00) with 10 participants; we therefore anticipated that the 9 participants we recruited would be a sufficient sample size to detect significant differences based on the power analysis. Athletes reported no musculoskeletal injuries at the time of data collection and provided written informed consent prior to participating in the study. The experimental protocol was approved by the University of Colorado Boulder Institutional Review Board (#18-0005).

### Experimental design

**Fig. 1.**
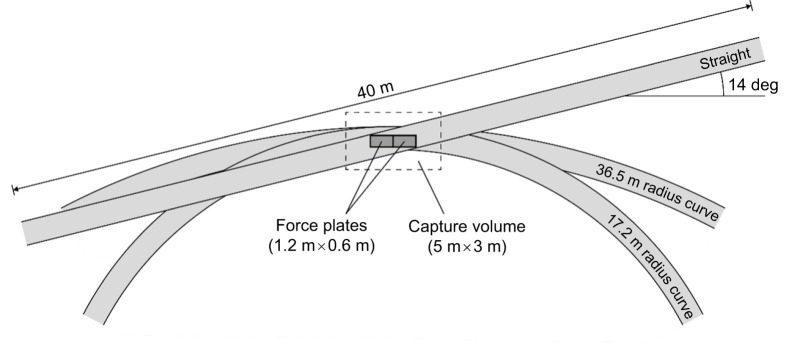
**Illustration of the experimental setup (to scale).** Two force plates and 10 motion capture cameras were located halfway along a 40 m straight section (‘straightaway’) and two curves (17.2 m and 36.5 m radii). Athletes adjusted their starting position on the curves to attain their perceived maximum sprinting velocity within the motion capture volume (dashed box). The straightaway was rotated 14 deg relative to the force plates to accommodate the constraints of the indoor track facility.

Athletes used their own spiked sprinting footwear and completed a randomized series of maximum effort sprints on a flat indoor Mondo-covered track (see below) over 1–2 days. Following a self-directed warm-up, athletes were instructed to perform maximum effort sprints on a 40 m straight section (‘straightaway’) and 40 m curves with radii of 17.2 m and 36.5 m in the CW and CCW directions. The order of trials was randomized for each subject. Each 40 m lane length and width were indicated with cones, and curve radii represented the innermost lane of a regulation flat 200 m and 400 m track (Meinel, 2008), respectively (Fig. 1). Athletes initiated each sprinting trial from a standing or crouched position, but no starting blocks were provided. Athletes practiced sprinting on the curves and adjusted their starting position to allow them to reach their perceived *v*_max_ halfway (∼20 m) along the straightaway or curve where we positioned two force plates flush with the track surface. Sprints were repeated for each condition until athletes successfully landed on a force plate with each leg at least once. We considered a trial to be unsuccessful if an athlete's foot was not entirely on the force plate during stance phase or they failed to stay within the lane of the curve (approximately 1.2 m width) for the entire 40 m. Data from all successful trials (231 steps) were used for analysis. Athletes were allowed ≥8 min of rest between trials to minimize any potential effects of fatigue.

### Materials

Two force plates (1000 Hz; 1.2×0.6 m; AMTI, Watertown, MA, USA) covered with an adhered track surface (Mondo S.p.A., Alba, Italy) were embedded in the ground so that the top surface was flush with the surrounding track surface and located halfway along the straightaway or curve. Ten motion capture cameras (200 Hz; 3×5 m capture volume; Vicon, Centennial, CO, USA) surrounded the force plates (Fig. 1). Prior to data collection, we adhered retroreflective markers onto each subject's pelvis and feet. Retroreflective markers on each foot were used to identify which leg was in contact with the force plate during a given trial and retroreflective markers on the pelvis were used to calculate sprinting velocity within the capture volume (Luo and Stefanyshyn, 2012a). We measured motion and GRFs simultaneously for each trial.

For all trials, we measured *v*_max_ from the capture volume using the average pelvis marker velocity, which was calculated using the retroreflective markers located bilaterally on the iliac crests, anterior superior iliac spines and posterior superior iliac spines. *v*_max_ was averaged over the length of the capture volume (∼5 m). Because of the location of the force plates in the indoor track facility, athletes were unable to adjust their starting position backwards on the straightaway to ensure that they attained *v*_max_ within the capture volume. Thus, we used a radar gun (47 Hz; Stalker ATS II, Applied Concepts Inc, Plano, TX, USA) on a tripod at a height of ∼1 m to measure velocity along the entire 40 m straightaway. To determine and verify *v*_max_ on the straightaway, we used the maximum value from a moving average of the radar gun velocity data (0.32 s window) and used this straight-running *v*_max_ to predict curve-running *v*_max_ for each mathematical model.

### Data processing

**Fig. 2.**
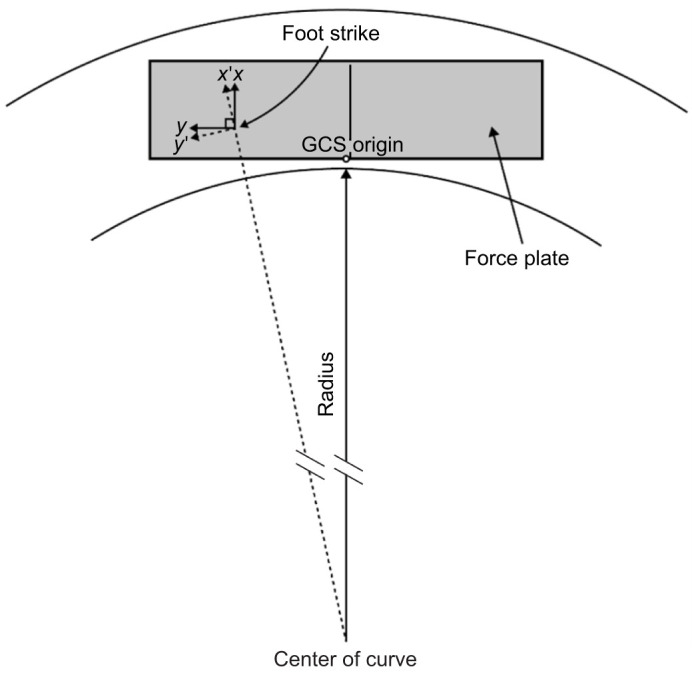
**Illustration of the transformed coordinate system.** *x*′ and *y*′ represent the transformed coordinate system of the force plate, represented by *x* and *y*, so that the centripetal horizontal axis was aligned perpendicular to the tangential horizontal axis relative to the position of the athlete on the curve. The coordinate system was rotated by the angle formed by the 3rd metatarsal head marker at the time of peak vertical ground reaction force (GRF), the center of the curve, and the origin of the global coordinate system (GCS).

We processed data using MATLAB (R2020a; MathWorks, Natick, MA, USA) with custom scripts and packages (Alcantara, 2019). 3D motion and GRF data were collected synchronously and filtered with a 4th order zero-lag low-pass Butterworth filter with a 50 Hz cutoff. We used a 5 N vGRF threshold to detect stance phase. Stance-average centripetal and vertical ground reaction force (cGRF_avg_ and vGRF_avg_) were calculated as the mean GRF during the stance phase for a given curve-running direction. We calculated stance average resultant GRF (rGRF­_avg_) for each trial as the vector sum of cGRF_avg_ and vGRF_avg_. To measure cGRF during the stance phase, we transformed the local coordinate system of the force plate so that the centripetal (mediolateral) horizontal axis was perpendicular to the tangential (anteroposterior) horizontal axis relative to the position of the athlete on the curve. For the curve conditions, this was accomplished by projecting the anterior–posterior and mediolateral horizontal GRFs relative to the force plate onto new coordinate system vectors rotated by the angle formed by the 3rd metatarsal head marker at the time of peak vGRF, the center of the curve, and the origin of the global coordinate system (Fig. 2). Across all trials, the transformed horizontal axes were rotated <3 deg from the force plate's original coordinate system. Because of the location of the force plates in the indoor track facility, athletes ran along a straightaway rotated 14 deg relative to the force plates (Fig. 1). Thus, we projected the anterior–posterior and mediolateral horizontal GRFs relative to the force plate on the straightaway onto a new coordinate system rotated by 14 deg.

To evaluate the curve-running *v*_max_ predictive models and assumptions, we calculated aerial time (*t*_a_), step time (*t*­_step_), swing time (*t*_swing_), step frequency and contact length (*L*_c_). All variables were calculated separately for the left and right legs. Most trials only included one step on a force plate, so we used the markers on each foot (right metatarsal head, left metatarsal head, and heel) in addition to the GRF to calculate these variables if they occurred immediately before or after an athlete contacted the force plate. For each subject, we used a 5 N vGRF threshold to calculate the average position of the foot markers and used the average position across trials to determine toe-off and heel-strike when athletes were not in contact with the force plate. We calculated *t*_a_ as the time from toe-off of one leg to heel-strike of the contralateral leg. We calculated *t*­_step_ as the sum of contact time (*t*_c­_) and the subsequent *t*_a_. We calculated *t*_swing_ as the sum of *t*_a_ and the subsequent *t*­_step._ Step frequency equaled the inverse of *t*­_step_. Finally, we determined *L*_c_­ as the total curved distance that the center of mass moved in the transverse plane from heel-strike to toe-off of the same foot using the average pelvis marker position. We created a model for each subject using their individual straight-running *v*_max_.

### Data analysis

We analyzed data using R (version 3.6.3) with custom scripts and packages (Wickham, 2009; https://CRAN.R-project.org/package=emmeans; https://CRAN.R-project.org/package=nlme; http://www.R-project.org/; https://CRAN.R-project.org/package=tidyr; https://CRAN.R-project.org/package=dplyr). We used a paired *t*-test (α=0.05) to compare our experimental data and the mathematical model predictions (equation 8.4 in McMahon, 1984; equation 11 in Greene, 1985; equation 2.9 in Usherwood and Wilson, 2005a,b) for how much *v*_max_ slows on a curve with a given radius relative to a straight path. In agreement with previous methods (Greene, 1985), we averaged data across trials for each condition and combined data from both sprinting directions when quantifying the changes in *v*_max_ on a curve relative to the straightaway and when comparing leg-specific kinematic variables (*L*_c_, step frequency and *t*_swing_) and GRF production across curve radii. We constructed linear mixed-effects models (LMEM) to quantify changes in *v*_max_, kinematic variables and GRFs across conditions. We considered condition (straight, 17.2 m radius curve, 36.5 m radius curve), leg relative to the center of the curve (inside, outside), and curve sprinting direction (CCW, CW) as categorical fixed effects and athlete as a random effect. Models were first constructed with interaction terms, but non-statistically significant model coefficients were removed from the model on the basis that the coefficient was not significantly different from zero. When statistically significant (*P*<0.05) interactions were present, we performed *post hoc* pairwise comparisons to analyze simple effects, applied the Bonferroni correction method to each family of comparisons, and reported the corrected α-value alongside the *P*-value. We also reported the numerical difference between each level of a fixed effect (e.g. inside versus outside leg) or the unstandardized model coefficients (*B*) alongside the *P*-value.

## RESULTS

### Velocity

**Fig. 3.**
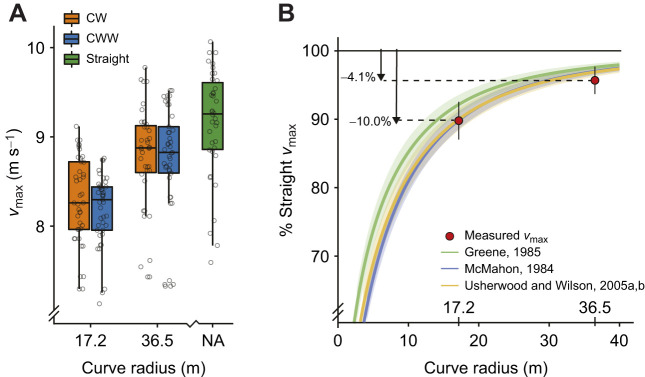
**Average curve and straight maximum sprinting velocity (***v*_max_**).** (A) Box plots showing the median, interquartile range, minimum and maximum *v*_max_ for all athletes (*n*=9) along the straightaway (green) and clockwise (CW; orange) or counterclockwise (CCW; blue) directions on the 17.2 m and 36.5 m curve radii. Gray circles show individual subject values. The linear mixed-effects model (LMEM) revealed that *v*_max_ decreased 10.0±2.4% (*P*<0.001) from the straightaway to the 17.2 m radius curve and 4.1±1.6% (*P*<0.001) from the straightaway to the 36.5 m radius curve (*P*<0.001) when combining data from both sprinting directions, and *v*_max_ was 1.6% faster in the CCW direction versus the CW direction (*P*=0.003). (B) Mean and standard deviation of the predicted percentage of straight-running *v*_max_ for a range of curve radii from mathematical models (Eqns 1, 2 and 3) from Greene (1985) (green), McMahon (1984) (blue) and Usherwood and Wilson (2005a,b) (orange) and measured mean and standard deviation of the percentage of straight-running *v*_max_ from the present study (red circles) where 100% is straight-running *v*_max_.

We found that the velocity measured from the radar gun during the straight-running trials captured *v*_max_ 2–10 m after athletes ran through the capture volume. Thus, we used the *v*_max_ measured from the radar gun for the straight-running trials and did not compare kinematic variables or GRFs between the straight- and curve-running conditions. We found that mean (±s.d.) *v*_max_ was 9.12±0.60 m s^−1^ for the straightaway and 8.21±0.44 m s^−1^ and 8.75±0.62 m s^−1^ for the 17.2 m and 36.5 m radius curves, respectively (Fig. 3A), when combining data from both sprinting directions. We found no interaction effect of sprinting direction and curve radius on *v*_max_ (*P*=0.122), indicating that the effects of curve radii and sprinting direction on *v*_max_ did not significantly depend on each other. The LMEM revealed that *v*_max_ decreased 10.0±2.4% (*P*<0.001) from the straightaway to the 17.2 m radius curve and 4.1±1.6% (*P*<0.001) from the straightaway to the 36.5 m radius curve (*B=*0.50 m s^−1^; *P*<0.001; Fig. 3) when combining data from both sprinting directions.

Using the mathematical models developed by McMahon (1984), Greene (1985) and Usherwood and Wilson (2005b), we calculated that regardless of sprinting direction, the *v*_max_ of athletes in the present study would slow by 9.3±1.2%, 6.8±1.4% and 9.2±1.3% from the straightaway to the 17.2 m radius curve, respectively, and slow by 1.9±0.4%, 1.0±0.5% and 2.0±0.3% from the straightaway to the 36.5 m radius curve, respectively. Thus, we found that the *v*_max_ prediction from McMahon (1984) was not significantly different from measured *v*_max_ for the 17.2 m radius curve (*P*=0.3) but overestimated *v*_max_ on the 36.5 m radius curve by 2.2% (*P*<0.01). We also found that the *v*_max_ prediction from Greene (1985) consistently overestimated *v*_max_ on the 17.2 m radius curve (*P*<0.005) and 36.5 m radius curve (*P*<0.005) by 3.0–3.5%. Finally, we found that the *v*_max_ prediction from Usherwood and Wilson (2005a,b) was not significantly different from measured *v*_max_ for the 17.2 m radius curve (*P*=0.2) but overestimated *v*_max_ on the 36.5 m radius curve (*P*<0.01) by 2.1%. Moreover, for a given curve radius, *v*_max_ in the CCW direction was 1.6% faster than that in the CW direction (*B*=0.14 m s^−1^; *P*=0.003; Fig. 3A).

### Kinematic variables

**Fig. 4.**
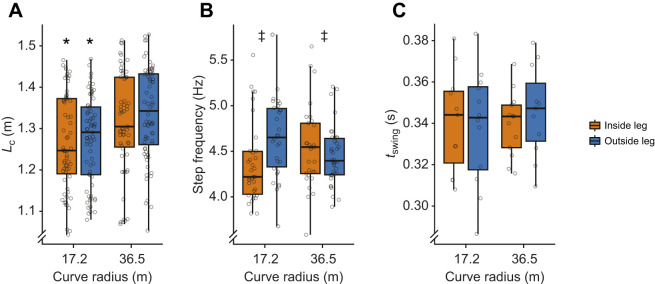
**Average kinematic variables at maximum speed for both curve-running directions.** Box plots showing the median, interquartile range, minimum and maximum of (A) contact length (*L*_c_, the total curved distance that the center of mass moved in the transverse plane from heel-strike to toe-off of the foot), (B) step frequency (the inverse of time from heel-strike to contralateral heel-strike) and (C) swing time (*t*_swing_, the time from toe-off to heel-strike of the same foot) for the inside (orange) and outside (blue) legs relative to the center of the curve for all athletes (*n*=9) while sprinting at maximum velocity on 17.2 m and 36.5 m curve radii curves. Gray circles show individual subject values. *Significant difference between curve radii (LMEM, *P*<0.05). ^‡^Significant difference between the inside and outside legs (LMEM, *P*<0.05).

We found that *L*_c_ was 0.06 m shorter when sprinting at *v*_max_ on the 17.2 m radius curve compared with the 36.5 m radius curve (*P*<0.001; Fig. 4A) when we combined data from both curve-running directions. We found no statistical difference in *L*_c­_ between the inside and outside leg or between running in the CW versus CCW direction. We found a significant interaction effect of curve radius and inside or outside leg on step frequency (*P*<0.05; Fig. 4B). Step frequency was 0.20 Hz lower for the inside leg compared with the outside leg on the 17.2 m radius curve (*P*<0.05; Fig. 4B) and was 0.03 Hz greater for the inside leg compared with the outside leg on the 36.5 m radius curve (*P*<0.05; Fig. 4B) for both curve-running directions. Additionally, we found no statistical difference in *t*_swing_ between curve radii, inside or outside legs, or sprinting direction (*P*>0.05). We did not compare kinematic variables between the straight and curved conditions because of athletes not reaching *v*_max_ within the capture volume for the straight conditions.

### rGRFs

**Fig. 5.**
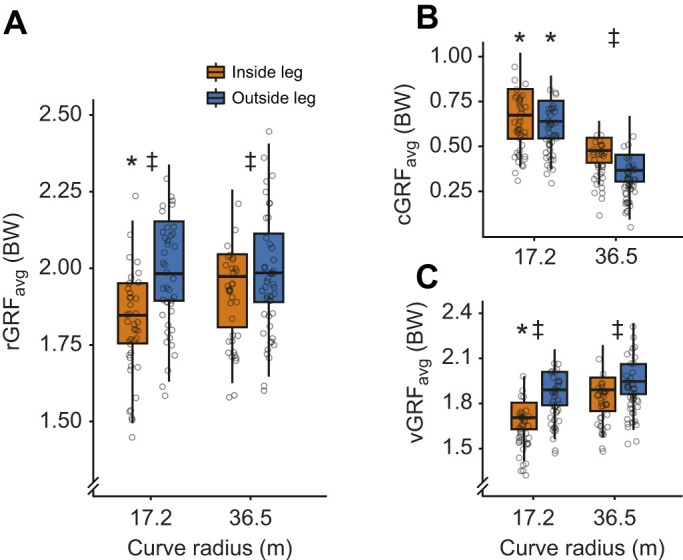
**Stance-average resultant, centripetal and vertical GRFs.** Box plots showing the median, interquartile range, minimum and maximum of stance average (A) resultant GRF (rGRF_avg_), (B) centripetal GRF (cGRF_avg_) and (C) vertical GRF (vGRF_avg_) (in body weight, BW) for the inside (orange) and outside (blue) leg relative to the center of the curve for all athletes (*n*=9). Gray circles show individual subject values. *Significant difference between curve radii per leg (LMEM, *P*<0.05). ^‡^Significant difference between the inside and outside legs (LMEM, *P*<0.05).

We averaged the rGRF_avg_ for both sprinting directions for the inside leg and the outside leg at each curve radius to compare inside versus outside leg rGRF_avg_ production. We found a significant interaction effect of curve radius and inside or outside leg on rGRF_avg_ (*P*=0.01; Fig. 5A). On the 17.2 m radius curve, we found that the rGRF_avg_ of the inside leg was 1.83 BW, which was 0.01 BW lower than that on the 36.5 m radius curve (*P*<0.001, α=0.0125; Fig. 5A). However, we found that the rGRF_avg_ of the outside leg was not significantly different (*P*=0.8, α=0.0125) between the 17.2 m (2.1 BW) and 36.5 m (2.0 BW) curve radii (Fig. 5A). Additionally, we found that the rGRF_avg_ of the inside leg was 0.18 BW (*P*<0.001, α=0.0125) and 0.11 BW (*P*=0.001, α=0.0125) lower than that of the outside leg on the 17.2 m and 36.5 m radius curves, respectively (Fig. 5A). We did not compare GRFs between the straight and curved conditions because of athletes not reaching *v*_max_ within the boundary of the capture volume for the straight conditions.

### cGRFs

We averaged the cGRF_avg_ for both sprinting directions for the inside leg and the outside leg at each curve radius to compare inside versus outside leg cGRF_avg_ production. We found a significant interaction effect of curve radius and inside or outside leg on cGRF_avg_ (*P*=0.029; Fig. 5B). On the 17.2 m radius curve, we found no statistically significant difference (*P*=0.089, α=0.0125; Fig. 5B) in cGRF_avg_ between the inside (0.68 BW) and outside legs (0.65 BW). On the 36.5 m radius curve, we found that the cGRF_avg_ of the inside leg was 0.48 BW, which was 0.10 BW greater than that for the outside leg (*P*<0.001, α=0.0125; Fig. 5B). On the 17.2 m versus 36.5 m radius curve, we found that cGRF_avg_ was 0.21 BW greater for the inside leg (*P*<0.001, α=0.0125) and 0.27 BW greater for the outside leg (*P*<0.001, α=0.0125; Fig. 5B).

### vGRFs

We averaged the vGRF_avg_ for both sprinting directions for the inside leg and the outside leg at each curve radius to compare inside versus outside leg vGRF_avg_ production. We found a significant interaction effect of curve radius and inside or outside leg on vGRF_avg_ (*P*=0.009; Fig. 5C). On the 17.2 m radius curve, we found that the vGRF_avg_ of the inside leg was 1.70 BW, which was 0.17 BW lower than that on the 36.5 m radius curve (*P*<0.001, α=0.0125; Fig. 5C). However, we found that the vGRF_avg_ of the outside leg was not significantly different (*P*=0.025, α=0.0125) between the 17.2 m (1.90 BW) and 36.5 m (1.97 BW) curve radii (Fig. 5C). Additionally, vGRF_avg_ of the inside leg was 0.21 BW (*P*<0.001, α=0.0125) and 0.10 BW (*P*=0.001, α=0.0125) lower than that of the outside leg on the 17.2 m and 36.5 m radius curve, respectively (Fig. 5C).

## DISCUSSION

In agreement with previous studies (Jain, 1980; Greene, 1985; Chang and Kram, 2007; Churchill et al., 2015, 2016; Taboga et al., 2016) and in support of our first hypothesis, we found that athletes had a 10.0% slower *v*_max_ on the 17.2 m radius curve and 4.1% slower *v*_max_ on the 36.5 m radius curve compared with that on the straightaway (Fig. 3B). We partially accept our second hypothesis that mathematical models would overpredict curve-running *v*_max_, as the *v*_max_ predictions from the mathematical models of McMahon (1984) and Usherwood and Wilson (2005b) were not statistically different from the measured *v*_max_ on the 17.2 m radius curve. The agreement between these predictions and the measured *v*_max_ may be due to the inclusion of kinematic variables in the equations. We found that the *v*_max_ predictions from the mathematical model of Greene (1985) were significantly different and consistently overestimated *v*_max_ on a given curve radius by 3.5% and 3.0% compared with measured *v*_max_ (Eqn 2) for the 17.2 m and 36.5 m radius curves, respectively. For the average *v*_max_ of the athletes in this study, a 3.5% overestimation is equivalent to a 0.3 m s^−1^ faster *v*_max_. Thus, if Greene's (1985) model was used to predict times in a 200 m race it would overpredict race time by 0.42 s, assuming an athlete is running at *v*_max_ on the straightaway and curve, and that half the race is on the curve. This may confirm that there is not a physiological limit to maximum rGRF (Chang and Kram, 2007) and supports the suggestion that values predicted through this mathematical model act as an upper bound to running performance (Greene, 1985). The mathematical models from McMahon (1984) and Usherwood and Wilson (2005b) (Eqns 1 and 3) overestimate *v*_max_ by 2.2% and 2.1% (0.19 s) for the 36.5 m radius curve, respectively.

We partially accept our third hypothesis that *L*_c_­, step frequency and *t*_swing_ would differ between curve radii and between the inside and outside legs. In agreement with previous studies, we found that *L*_c_ was 4.7% shorter at *v*_max_ on the 17.2 m compared with the 36.5 m radius curve (Fig. 4A) (Chang and Kram, 2007; Taboga et al., 2016; Churchill et al., 2015). All three of the mathematical models that predict curve-running *v*_max_ assumed that *L*_c_ was independent of curve radius ­(McMahon, 1984; Greene, 1985; Usherwood and Wilson, 2005b). Thus, accounting for the changes in *L*_c_ across curve radii may be needed to improve curve-running *v*_max_ predictions. We found that step frequency was independent of curve radius, which supports the assumptions of the models proposed by McMahon (1984) and Greene (1985). However, we also found that step frequency was 4.7% faster for the outside leg compared with the inside leg on the 17.2 m radius curve, and 0.7% slower for the outside leg compared with the inside leg on the 36.5 m radius curve (Fig. 4B). Thus, it may be necessary to account for leg-specific differences in step frequency on different curve radii to improve curve-running *v*_max_ predictions. We found that *t*_swing_ did not change between the 17.2 m and 36.5 m curve radii (Fig. 4C) or between legs. Our results thus support the assumption of Usherwood and Wilson (2005b) that *t*_swing_ does not depend on curve radius or on the inside versus outside leg.

We reject our fourth hypothesis that rGRF_avg_ would not change between curve radii or between the inside and outside leg because we found a 5% decrease in rGRF_avg_ between the 36.5 m and 17.2 m radius curves (Fig. 5A) and that the inside leg produced lower rGRF_avg_ than the outside leg at *v*_max_ on both curve radii. Our findings are in line with those of Chang and Kram (2007), who found that maximum rGRF at *v*_max_ on small radii (1–6 m) was lower than that on a straight track, and maximum rGRF decreased with a decreasing curve radius. These findings suggest that curve sprinting *v*_max_ may not be limited by a physiological limit to maximum rGRF. We suggest that the decrease in rGRF_avg_ may be due to other physiological constraints such as the kinematic configuration of the lower limb segments while sprinting around the curve. For both curve radii at *v*_max_, we found that the inside leg consistently produced lower rGRF_avg_ than the outside leg. These findings refute the underlying assumption that there is no difference between the inside and outside legs that is used in all three mathematical models that predict curve-running *v*_max_ (McMahon, 1984; Greene, 1985; Usherwood and Wilson, 2005b). Additionally, our findings agree with previous studies that found that on small curve radii (1–6 m), the inside leg produces lower maximum cGRF and vGRF than the outside leg (Chang and Kram, 2007), but on larger curve radii (37.72 m), the inside leg produces greater maximum cGRF than the outside leg but similar maximum vGRF (Churchill et al., 2016).

We partially accept our fifth hypothesis that the inside leg would produce greater cGRF_avg_, but lower vGRF_avg_, than the outside leg while sprinting at *v*_max_ on both curve radii. The greater cGRF_avg_ produced by the inside versus outside leg on the 36.5 m radius curve is consistent with previous studies that investigated leg-specific cGRF on a curve radius of 37.72 m during maximum effort sprinting (Churchill et al., 2016; Judson et al., 2019). However, previous studies have shown that the outside leg produced greater cGRF than the inside leg during maximum effort sprinting on curve radii ≤6 m, similar to performing a lateral cutting maneuver (Rand and Ohtsuki, 2000; Chang and Kram, 2007). We found that there was no significant difference in cGRF_avg_ between the inside and outside legs on the 17.2 m radius curve (Fig. 5B). These findings suggest a potential transition where the cGRF_avg_ produced by the outside leg exceeds that produced by the inside leg to navigate curves with smaller radii and may partially explain differences in results for the inside and outside legs from studies that collected cGRF on smaller (1–6 m) and larger (37.72 m) curve radii.

In support of our fifth hypothesis, we found that vGRF_avg_ was 0.10–0.21 BW greater for the outside compared with the inside leg for both curve radii (Fig. 5C). vGRF and cGRF production and thus sprinting performance are due in part to the force produced by the ankle plantarflexor muscles (Dorn et al., 2012; Luo and Stefanyshyn, 2012a; Nagahara et al., 2018), and leg-specific frontal plane ankle inversion and eversion may limit the ability of ankle plantarflexor muscles to generate cGRF and vGRF during *v*_max_ curve sprinting. The production of cGRF and vGRF may differ between the inside and outside legs as a result of differences in the peak ankle plantarflexor moment (Judson et al., 2020a) and peak ankle eversion angle (Alt et al., 2015) during maximum effort curve sprinting, but further research is needed to investigate the effect of curve radii on leg-specific joint kinetics, joint kinematics and GRF production. Athletes seeking to improve curve sprinting performance may benefit from strengthening ankle plantarflexor muscles under a range of frontal plane ankle orientations, as maximum ankle inversion and eversion angles significantly differ during curve versus straight sprinting (Alt et al., 2015; Judson et al., 2020b).

Our findings support our sixth hypothesis that *v*_max_ would be slower in the CW versus CCW direction. We found that athletes had 1.6% slower *v*_max_ in the CW compared with the CCW direction regardless of the curve radius. This effect of sprinting direction on *v*_max_ is similar to the results of Taboga et al. (2016), who found that athletes had 1.9% slower *v*_max_ in the CW compared with the CCW direction on a curve with a 17.2 m radius (Taboga et al., 2016). We suspect these results are due to athletes' familiarity of sprinting in the CCW direction for competitions and the potential differences in strength between the inside and outside legs, but we did not investigate these potential effects. Future studies are warranted to determine whether there are muscle strength differences between the inside and outside legs of competitive 200 m and 400 m sprinters.

One of the potential limitations of our study to consider alongside our findings is that we used a radar gun to measure *v*_max_ on the straightaway and 3D motion capture data to measure *v*_max_ on the curves. This approach was necessary because the mathematical models depend on straight-running *v*_max_ and athletes were unable to adjust their starting position on the straightaway to ensure they reached *v*_max_ within the capture volume because of the constraints of our indoor track facility. Despite measuring *v*_max_ on the straightaway and curves using different methods, both provide accurate and consistent measures of running velocity (Chelly and Denis, 2001; di Prampero et al., 2005; Morin et al., 2006; Luo and Stefanyshyn, 2012b; Zrenner et al., 2018). Further analysis of the radar gun data revealed that *v*_max_ was achieved 2–10 m beyond the boundary of the capture volume (Fig. 1). *v*_max_ measured from the radar gun exceeded the velocity measured in the capture volume by 0.44±0.2 m s^−1^. We also found that the velocity measured by the radar gun within the capture volume was not significantly different from the velocity calculated from the 3D motion capture data (paired *t*-test, *P*=0.582). Additionally, to determine whether athletes were at a constant velocity on the force plate, we compared the anterior–posterior horizontal propulsive impulse with the braking impulse, where the impulse was calculated as the integral of force with respect to time, propulsive impulse was the positive impulse and braking impulse was the negative impulse. We found that the horizontal propulsive impulse was on average 0.04 N s greater than the braking impulse when athletes ran on the straightaway (paired *t*-test, *P<*0.05) over the force plates, indicating that athletes were accelerating on the straight path. However, there was no difference between propulsive and braking impulses during running on the curves (paired *t*-test, *P*=0.3). Therefore, we assume that athletes were neither accelerating nor decelerating and likely running at their *v*_max_ for each curve-running trial. Because athletes did not achieve *v*_max_­ on the force plate during the straight-running trials, we did not statistically compare GRFs or kinematic variables between the straight-running and curve-running trials (Table S1). Although we did not compare curve-sprinting vGRF production with straight-running vGRF production, previous work investigating submaximal straight and curve (36.5 m radius) sprinting found that there was no significant difference in peak vGRF between straight and curve sprinting for either leg (Viellehner et al., 2016). Moreover, we used the position of the metatarsal foot markers to determine the coordinate system for the cGRF and found that it differed with the orientation of the force plates by <3 deg. If we did not correct for this angle change, the difference in cGRF_avg_ would have been small. For example, if the horizontal forces were offset by 3 deg, this would change cGRF_avg_ by 0.003 N on a 36.5 m curve radius. Lastly, we combined data from the CW and CCW sprinting directions when investigating the effect of curve radius and the inside or outside leg on GRF production, which assumes that there are no anatomical asymmetries between the legs such as differences in muscle strength. We suspect that the slower *v*_max_ for the CW versus CCW direction may be due to a trained sprinter's unfamiliarity with CW sprinting, but future work should investigate the potential biomechanical mechanisms such as strength differences between legs that could be responsible for the differences we observed in CW and CCW *v*_max_.

Chang and Kram (2007) found that on curves with small radii (1–6 m), *v*_max_ was not constrained by maximum limb force generation of the inside or outside leg and suggested that a combination of different biomechanical constraints led to the inside leg limiting the generation of forces necessary to achieve similar *v*_max_ on curves compared with a straight track. Additionally, when exploring the attenuation of *v*_max_ on curves in horses, Tan and Wilson (2011) showed that at small curve radii, *v*_max_ is likely limited by friction, whereas at larger curve radii, the slowing of *v*_max_ is likely due to a limit in maximum limb force generation. Coupling these previous studies with our findings suggests that limitations in curve-sprinting *v*_max_ change with curve radii and may be due to different biomechanical mechanisms such as cGRF and vGRF production differences between legs for different curve radii. Additionally, previous research suggests that the *v*_max_ of greyhounds does not slow on curves compared with a straight path because of the mechanical separation of the muscles that provide power from the structures that support body weight (Usherwood and Wilson, 2005a). However, in humans, the lower limb muscles generate power and support body weight. Moreover, the position of the lower limb may affect force production. Future studies are needed to determine how lower limb joint moments and power affect maximum effort sprinting performance on a curve.

### Conclusions

We determined how *v*_max_ changes on two flat regulation track curves compared with a straight track and the kinematics and GRFs produced by the inside and outside legs. We found that, compared with that for a straight track, *v*_max_ slowed by 10.0% and 4.1% on a 17.2 m and 36.5 m radius curve, respectively. We compared these results with predictions from mathematical models and found that the *v*_max_ predicted by models proposed by McMahon (1984) and Usherwood and Wilson (2005b) were not different compared with the measured *v*_max_ on the 17.2 m radius curve; however, both mathematical models overpredicted *v*_max_ by 2.1–2.2% on the 36.5 m radius curve. The *v*_max_ predicted by the model proposed by Greene (1985) overestimated *v*_max_ on the 17.2 m and 36.5 m radius curves by 3.0–3.5%. We tested the four main assumptions used when developing the mathematical models and found that *L*_c_ was not independent of curve radius and decreased with a smaller curve radius. Additionally, we found that step frequency and *t*_swing_ did not change between 17.2 m and 36.5 m radius curves. Moreover, we found that rGRF_avg_ decreased between the 36.5 m and 17.2 m radius curves. Thus, future predictive models of maximum curve-running velocity should account for differences in *L*_c_ and rGRF_avg_ with changes in curve radius. We also found that sprinters modulate the rGRF, cGRF and vGRF produced by their inside and outside legs as curve radius decreases. Limitations to leg-specific cGRF and vGRF production may be due to frontal plane ankle kinematics of the inside and outside legs during maximum effort curve sprinting. Future studies are needed to better understand leg-specific joint kinematics and kinetics during maximum effort curved sprinting and their influence on performance.

## Supplementary Material

10.1242/jexbio.246649_sup1 Supplementary information
