# Supplementary material for: Maximum velocity and leg-specific ground reaction force production change with radius during flat curve sprinting
Source: J Exp Biol. 2024 Feb 28;227(4):jeb246649. doi: 10.1242/jeb.246649 (PMC11093109; doi:10.1242/jeb.246649)
Supplement: Supplementary information [file jexbio-227-246649-s1.pdf]

Table S1. Kinematic and Kinetic Variables for the Curved and Straightaway conditions

	**17.2m radius**		**36.5m radius**		**Straightaway**
Variable	Inside Leg	Outside Leg	Inside Leg	Outside Leg	
**Capture Volume Velocity (m/s)**	8.24 ± 0.44		8.75 ± 0.61		8.76 ± 0.60
**Vertical Ground Reaction Force (BW)**	1.70 ± 0.16	1.90 ± 0.15	1.87 ± 0.14	1.97 ± 0.18	1.90 ± 0.16
**Centripetal Ground Reaction Force (BW)**	0.69 ± 0.15	0.65 ± 0.13	0.48 ± 0.11	0.38 ± 0.11	______
**Resultant Ground Reaction Force (BW)**	1.83 ± 0.19	2.01 ± 0.18	1.94 ± 0.15	2.01 ± 0.19	______
**Contact Length (m)**	1.26 ± 0.12	1.28 ± 0.10	1.32 ± 0.122	1.34 ± 0.11	1.35 ± 0.11
**Step Frequency (Hz)**	4.37 ± 0.44	4.65 ± 0.43	4.55 ± 0.45	4.45 ± 0.312	4.50 ± 0.48
**Swing Time (s)**	0.34 ± 0.02	0.34 ± 0.03	0.34 ± 0.02	0.35 ± 0.02	0.34 ± 0.03

Journal of Experimental Biology • Supplementary information
